# De la Luxation congénitale du Fémur

**Published:** 1897-09

**Authors:** 


**De la Luxation congénitale du Fémur.** Par le Dr. Édouard Delanglade. Pp. 332. Paris: G. Steinheil. 1896.

This is the best and most complete monograph on the subject of congenital dislocation of the hip that we have seen. The author has devoted a considerable amount of study to the literature of the subject, and has evidently had a very considerable experience in dealing with these cases, and he gives us here a valuable digest of his work, and very ably sums up the whole subject.

Dr. Delanglade shows beyond all doubt that the cause of the affection is an original malformation, and that it is not due, as was once supposed, to an injury at the time of birth. The cotyloid cavity is always found rudimentary and incapable of retaining the head of the femur in its cup. The muscles also are imperfectly developed, being not only less in volume and consistence to the naked eye, but containing histologically a diminution in their muscular fibrillæ. Obstacles to the reduction of these dislocations are of three varieties, osseous, ligamentous, and muscular, and the author enters fully into the best methods of overcoming all these difficulties. He enumerates the various operations which have been devised for the relief of these affections and divides them into orthopædic or bloodless operations, and cutting operations, giving the preference in most cases to the latter method, but pointing out that in young patients up to three years of age, and especially in unilateral cases, great success may be obtained by the orthopædic method, especially if the directions of Lorenz are closely followed. The book is enriched by particulars of 133 cases, taken from various sources, full particulars of the treatment carried out being given, and in many cases the results of *post-mortem* examinations are added. A very complete bibliography is given at the end of the book.

